# Supplementary material for: Discovery of Small Molecules That Inhibit MYC mRNA Translation Through hnRNPK and Induction of Stress Granule-Mediated mRNA Relocalization
Source: Int J Mol Sci. 2025 Aug 22;26(17):8139. doi: 10.3390/ijms26178139 (PMC12427919; doi:10.3390/ijms26178139)
Supplement: Supplementary file 1 [file ijms-26-08139-s001.zip › ijms-3803298-supplementary.pptx]

## Slide 1
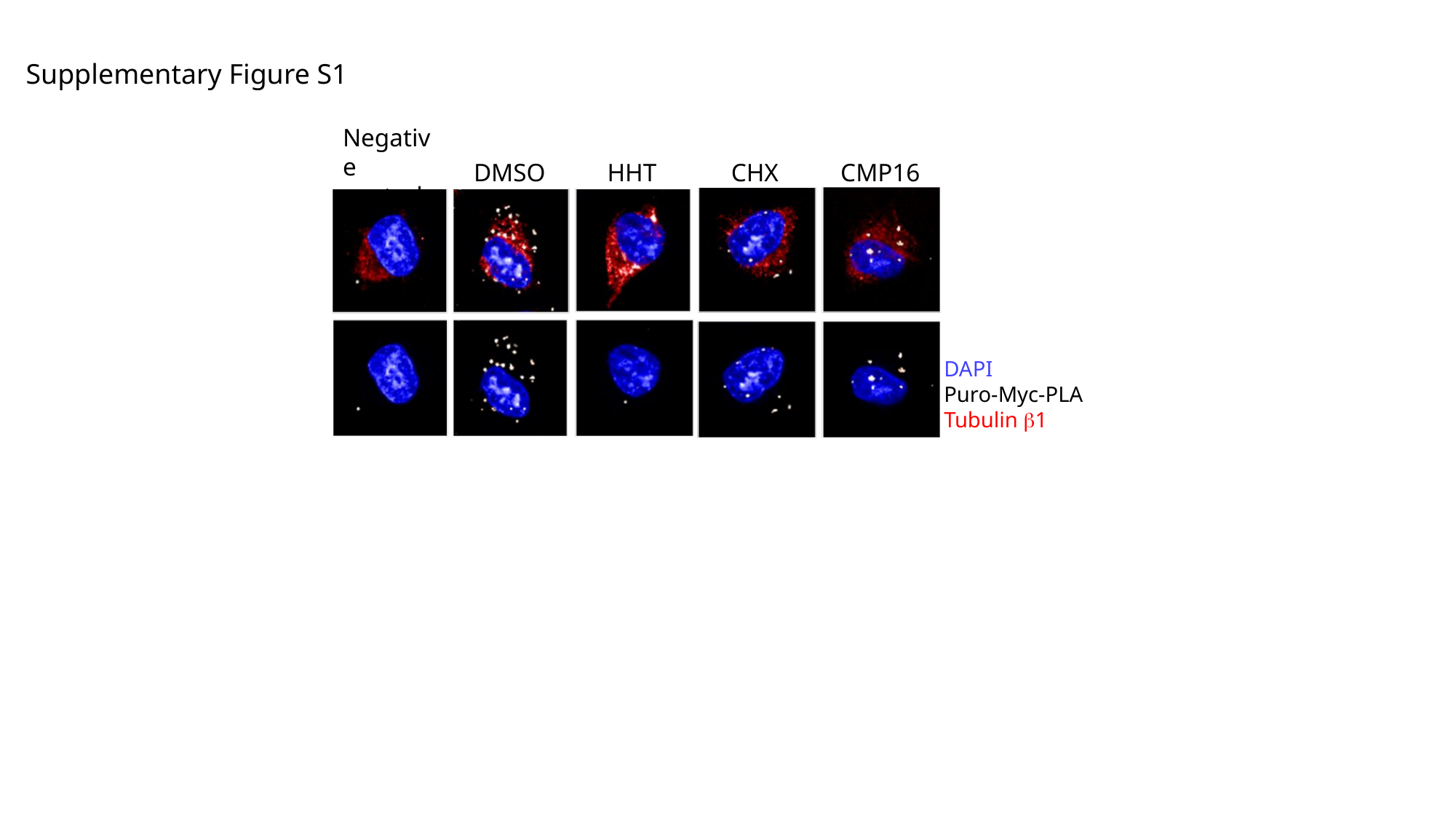

Supplementary Figure S1
Negative control
DMSO
HHT
CHX
CMP16
DAPI
Puro-Myc-PLA
Tubulin b1

## Slide 2
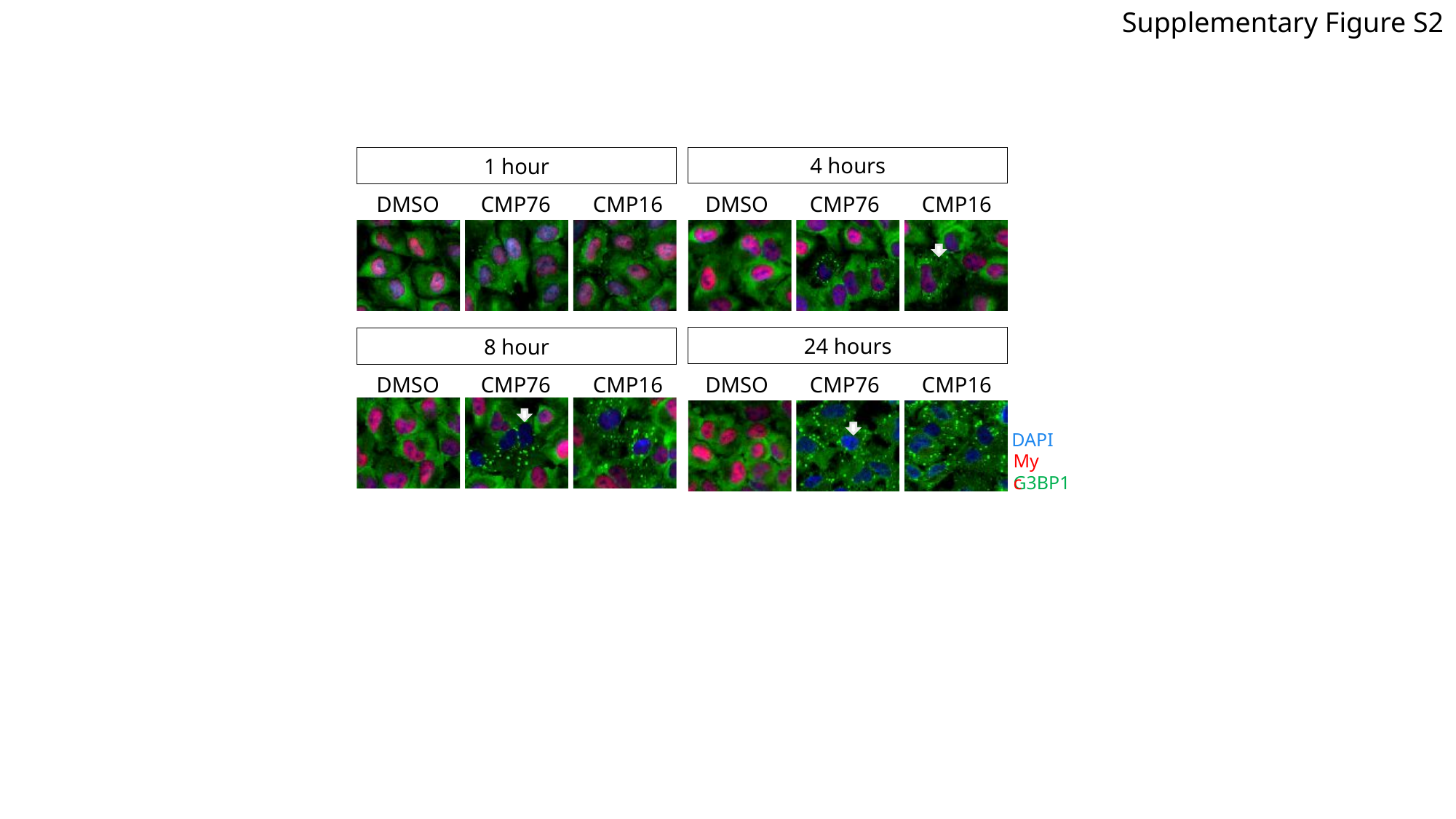

Supplementary Figure S2
4 hours
1 hour
DMSO
CMP76
CMP16
DMSO
CMP76
CMP16
24 hours
8 hour
DMSO
CMP76
CMP16
DMSO
CMP76
CMP16
DAPI
Myc
G3BP1
